# Soil bacterial communities of paddy are dependent on root compartment niches but independent of growth stages from Mollisols of Northeast China

**DOI:** 10.3389/fmicb.2023.1170611

**Published:** 2023-04-14

**Authors:** Kai Liu, Qiuju Wang, Minglong Sun, Shiwei Gao, Qing Liu, Lili Shan, Junxiang Guo, Jingyang Bian

**Affiliations:** ^1^Heilongjiang Academy of Agricultural Sciences, Harbin, China; ^2^Heilongjiang Academy of Black Soil Conservation and Utilization, Harbin, China; ^3^Crop Resources Institute, Heilongjiang Academy of Agricultural Sciences, Harbin, China; ^4^Suihua Branch of Heilongjiang Academy of Agricultural Sciences, Suihua, China; ^5^Rice Research Institute of Heilongjiang Academy of Agricultural Sciences, Jiamusi, China; ^6^Daqing Branches of Heilongjiang Academy of Agricultural Sciences, Daqing, China

**Keywords:** bacterial microbiome, distribution, MiSeq sequencing, co-occurrence network, rice fields

## Abstract

**Introduction:**

Deep insights into adhering soil of root zones (rhizosphere and rhizoplane) microbial community could provide a better understanding of the plant-microbe relationship. To better understand the dynamics of these microbial assemblies over the plant life cycle in rhizodeposition along rice roots.

**Methods:**

Here, we investigated bacterial distribution in bulk, rhizosphere, and rhizoplane soils at tillering, heading, and mature stage, from rice (*Oryza sativa*) fields of the Northeast China.

**Results and Discussion:**

Our results revealed that soil bacterial α-diversity and community composition were significantly affected by root compartment niches but not by temporal change. Compared to rhizoplane soils in the same period, bulk in the heading and rhizosphere in the mature had the largest increase in Shannon’s index, with 11.02 and 14.49% increases, respectively. Proteobacteria, Chloroflexi, Bacteroidetes, and Acidobacteria are predominant across all soil samples, bulk soil had more phyla increased across the growing season than that of root related-compartments. Deterministic mechanisms had a stronger impact on the bacterial community in the compartments connected to the roots, with the relative importance of the bulk soil, rhizoplane and rhizosphere at 83, 100, and 56%, respectively. Because of ecological niche drivers, the bacterial networks in bulk soils exhibit more complex networks than rhizosphere and rhizoplane soils, reflected by more nodes, edges, and connections. More module hub and connector were observed in bulk (6) and rhizoplane (5) networks than in rhizosphere (2). We also detected shifts from bulk to rhizoplane soils in some functional guilds of bacteria, which changed from sulfur and nitrogen utilization to more carbon and iron cycling processes. Taken together, our results suggest distinct bacterial network structure and distribution patterns among rhizosphere, rhizoplane, and bulk soils, which could possibly result in potential functional differentiation. And the potential functional differentiation may be influenced by plant root secretions, which still needs to be further explored.

## Introduction

In nature, plant roots associate with several soil-derived bacterial microbiota, which impact plant development, nutrient uptake, and disease resistance positively or negatively (Edwards et al., 2018; Dang et al., 2022; Tspa et al., 2022). Previous studies using next-generation sequencing technologies have shown that root microbiota composition is predominantly controlled by environmental factors, plant genotype and soil type (Dang et al., 2022). In fact, due to the selective pressure imposed by the environment, which results in exchanges within a local population and migrations between other populations, microbial populations can experience brief alterations in their structure (Cavaglieri et al., 2009). It is well known that plant development has an impact on the production and diffusion of root exudates (Canarini et al., 2019). These exudates secreted by plants stimulate specific microorganisms, and the function of exudates is also influenced by the stage of plant growth (Wen et al., 2021). Therefore, the degree of adaptation of soil microorganisms may vary with the different stages of plant development.

Four distinct compartments from the outside to the inside of the root: bulk soil (unaffected by root activity), rhizosphere (the soil microenvironment immediately surrounding the root), rhizoplane (the root surface), and endosphere (the root interior), and microbial diversity follows a compositional transition along this gradient (Van der Heijden and Schlaeppi, 2015). Since root-associated microorganisms are mostly sourced from the local edaphic communities, the specific compositions of these compartments rely on the soil source. Roots maintain a complex microbial community at the soil-root interface, which can affect plant nutrition, growth, and health (Yang H. J. et al., 2022). Huang et al. found significant differences in the structure and function of bacterial communities among the three root-associated ecological niches (bulk soil, rhizosphere, and rhizoplane), such as bacterial community diversity and composition (Huang et al., 2022). Rhizocompartment were the dominant factors affecting bacterial assemblage, with bacterial community OTU numbers decreasing from bulk soil to rhizoplane and specific OTUs enriching from bulk soil to rhizoplane (Lang et al., 2019). In addition, Yamazaki et al. also pointed out that bacterial communities in rhizosphere environments differ from those in bulk soils through a multi-omics analysis (microbiome and transcriptome) of soybean plants in a field at Tokyo University of Agriculture and Technology, Japan (Yamazaki et al., 2021). However, most studies have focused on studying the effects of rhizocompartment on bacterial communities without considering the effects of plant life cycles and the interaction between the two on soil bacterial communities (Lucas et al., 2018; Yin and Yan, 2020; Li B. et al., 2022). Deterministic processes based on ecological niches and neutral stochastic processes are two processes that explain the changes in microbial community assembly, which may be influenced by the external environment (Beck et al., 2015; Zhou and Ning, 2017). Similarly, there are more and more studies to promote soil microbial interactions by constructing correlation network analysis (Wang et al., 2019; Xiao et al., 2022; Li et al., 2023). For example, by exploring the effect of tillage practices on soil bacterial communities, Liu et al. (2021) found that deep tillage leads to a tighter and more competitive network of better soil bacterial communities. Because of this, we attempted to construct microbial network analysis and community assembly to investigate the mechanisms of soil bacterial communities affected by rhizocompartment under different rice life cycles.

More than 65% of Chinese people use rice as their primary food source, and an area of 3 × 10^7^ ha represents 20% of the total world cropping area (FAO, 2019). Consequently, it is crucial to investigate the changes of microorganisms in root environment of rice. The objective of the present study was to focus on two main questions (1) How variable are bacterial communities associated with different plant growth stages? and (2) Whether there are spatial variations in the soil rhizodeposition. We offer insights into the process of rice root bacteria development through dynamic investigations of the composition of the rice bacteria.

## Materials and methods

### Field experiment design

The experimental site was located at the experimental field of the Heilongjiang Academy of Agricultural Sciences in Minzhu village, Harbin, China (45°49′N, 126°50′E). The area has a temperate continental monsoon climate. The mean annual temperature in the region is 3.6°C, with a frost-free period of 141 days. The mean annual rainfall is 502 mm, and nearly 60% of the total rainfall is mainly concentrated from July to September. The mean annual precipitation is 1,032.5 mm (Li et al., 2019). The soil was a clay loam (Mollisols Udolls Paleudolls) according to the USDA soil Taxonomy system (Soil Survey, 2010). The soil properties are listed: organic matter, 35.1 g·kg^−1^; total N, 1.42 g·kg^−1^; total P, 0.38 g·kg^−1^; total K, 21.37 g·kg^−1^; available N, 148.2 mg·kg^−1^; available P, 38.5 mg·kg^−1^; available K, 249.1 mg·kg^−1^; and pH 6.53.

A susceptible rice variety, Wuyoudao-4 was planted on March 16 and harvested on October 20 in 2019 using approximately 30 cm seedlings at an inter-row spacing of 0.20 m. The planting densities were maintained at 280,000 plants ha^−1^. Three replicates of the treatments were set up in a randomized block design, with each plot size 5 m × 4.5 m each and being spaced apart by 2 m. During the growing season, weeds were manually pulled twice; no herbicide or rhizobium inoculant was used. Nitrogen fertilization (140 kg N ha^−1^) was supplied as urea on June 7, 2019 and June 30, 2019, respectively.

### Sample collection

Rice plants (*n* = 10–12) were harvested by digging around for maximum intactness of the roots at each site. This study has 3 replications for each treatment. Soil sampling procedure and compartment separation were described by Edwards et al. (2015) (Supplementary Table S1). Specifically, soil and root sample collection were at tillering, heading and mature stages, respectively. The sampling times are listed: July 8 (tillering stage), August 1 (heading stage), and September 25, 2019 (mature stage). All soil samples were placed in polyethylene bags and sent to the lab on ice packs. The soils were hand-selected to remove small stones, residues, and roots, and then sieved through 2 mm meshes. Storage of the samples was at −80°C for DNA extraction.

### Sequencing of the 16S rRNA gene

The total DNA of each sample was isolated from 0.25 g of soils using the PowerSoil DNA Isolation Kit (MoBio Laboratories, Inc., Carlsbad, CA, United States). Prior to amplification, the DNA concentration was standardized to be the same. The 16S rRNA gene’s V3-V4 region was chosen to create the community library using the forward primers 338\u00B0F (Huse et al., 2008) and reverse primer 806 R (Li et al., 2019), using a six-base barcode that is particular to each sample. Supplementary Table S2 offers more information on the PCR conditions. PCR products from all samples were combined and identified by 2% agarose gel electrophoresis, and the PCR products were recovered by gel cutting using the AxyPrep DNA Gel Recovery Kit (AXYGEN). PCR products were quantitatively detected by QuantiFluor™-ST Blue fluorescence quantitative system (Promega), and the MiSeq sequencing library was constructed using TruSeqTM DNA Sample Prep Kit, which was sequenced based on PE300 strategy. The PCR products were purified, pooled in equimolar amounts, and paired-end sequenced (2 × 300) using an Illumina MiSeq platform at Shanghai Meiji Biological Medicine Technology Co Ltd., Shanghai, China.

### Bioinformatics analysis

Low-quality raw data sequences (length < 250 bp and average base quality score < 20) were removed using Trimmomatic (Bolger et al., 2014) and merged using FLASH software (Magoc and Salzberg, 2011; Bolger et al., 2014). In total, 1,460,755 high-quality reads were generated in this study. Using the USEARCH v7.1 pipeline, operational taxonomic units (OTUs) were produced with at a ≥ 97% similarity level (Edgar, 2013), which yielded 6,513 OTUs. The remaining sequences were denoised (Schloss et al., 2009) and aligned against the bacterial 16S rRNA gene database in Mothur (Koljalg et al., 2013). The 16S rRNA gene sequences were submitted to the Sequence Read Archive (SRA) at the National Center for Biotechnology Information (NCBI) with accession number PRJNA738275.

### Statistical analysis

Based on this output normalized data, subsequent analyzes of alpha and beta diversity were all carried out. Alpha-diversity indices were calculated with Mothur (version1.30.2). The effect of rhizospheric compartmentalization, temporal change, and their interaction on soil bacterial community composition was assessed using Permutational multivariate analysis of variance (PERMANOVA). Subsequently, beta-diversity of bacterial community was ordinated using principal coordinates analysis (PCoA). Beta regression was performed using the BetaReg package (Cribari-Neto and Zeileis, 2010). In order to evaluate the community assembling processes, a null modeling approach was used to calculate the beta Nearest Taxon Index (NTI) (Stegen et al., 2013). The βNTI between −2 and 2 range denotes stochasticity, while theβ NTI > 2 or < −2 indicates determinism in community assembly (Dini-Andreote et al., 2015). Additional, based on both βNTI and Bray–Curtis-based Raup-Crick Index (RC_Bray_) values, five ecological processes were examined: homogeneous selection (βNTI < −2), heterogeneous selection (βNTI > +2), dispersal limitation (βNTI <2 and RC_Bray_ > 0.95), homogenizing dispersal (βNTI <2 and RC_Bray_ < −0.95), and undominated (βNTI <2 and RC_Bray_ < 0.95) were analyzed (Zhou et al., 2014; Jiao et al., 2020). To better comprehend the taxonomic and functional relationships within the microbial communities, network studies were carried out (Mendes et al., 2014). Bacterial OTUs that appeared in over 50% of the communities were included in the networks analysis so that we could concentrate exclusively on the abundant OTUs (Guan et al., 2020). Spearman’s correlation coefficients were calculated between OTUs (Revelle, 2013). The correlations between OTUs with a Spearman’s coefficient < 0.7 and a *p* value >0.01 were deleted in order to filter the data for reduced network complexity (Widder et al., 2014). Network visualisation was done using igraph package Csardi and Nepusz (2006). Then, in each network, the threshold values of Zi and Pi as given by Guimera and Amaral (2005) were used to evaluate the topological roles of each node. The bacterial functional groups were evaluated using Functional Annotation of Prokaryotic Taxa (FAPROTAX) after obtaining the OTU’ identification and abundance information (Sansupa et al., 2021). All samples’ distribution of function groups was evaluated using the PCA and heatmap. The analyzes indicated above were completed using R program (v.3.1.1) (R Core Team, 2013).

Two-way ANOVAs were used to assess the effects of rhizospheric compartmentalization, temporal change, and their interaction on soil bacterial diversity and richness indices. Statistical differences among rhizospheric compartmentalization and temporal change were measured by Tukey’s HSD and Mann–Whitney non-parametric test with subsequent Bonferroni correction, with *p* < 0.05 considered significant.

## Results

### Soil bacterial diversity index

Soil bacterial Shannon diversity, Simpson diversity, Ace richness, and Chao 1 richness indices were significantly affected by rhizospheric compartmentalization but not by temporal change and their interaction (Supplementary Table S3). The Shannon index was significantly higher in bulk and rhizosphere soils (increased by 3.73 and 8.24% in the tillering, 11.09 and 12.19% in the heading, and 8.97 and 14.58% in the mature stage, respectively). However, the differences between bulk and rhizoplane soil were not significant (*p* > 0.05) in the tillering stage. The results of the Simpson index showed the opposite trend to that of the Shannon index only in heading stage (Table 1). The Ace and Chao 1 indices show the same trend; the Ace and Chao 1 indices were significantly higher in bulk (11.54 and 12.28%, respectively) and rhizosphere soils (17.14 and 16.86%, respectively) than that of rhizoplane soil in the heading stage; in the mature stage, rhizosphere soils showed an increase of 14.95 and 19.71% in Ace and 15.68 and 20.26% in Chao 1 indices compared with bulk and rhizoplane soils, whereas Ace and Chao 1 indices were not shown significantly differences among rhizospheric compartmentalization (*p* > 0.05) in the tillering stage.

**Table 1:** Soil bacterial Shannon diversity, Simpson diversity, Ace richness, and Chao 1 richness indices among three compartments in the tillering, heading, and mature stage.

		Shannon	Simpson	Ace	Chao 1
Tillering	BS	6.67 ± 0.23 ab	0.0044 ± 0.0010 a	3103.93 ± 322.85 a	3117.76 ± 290.76 a
	R1	6.96 ± 0.14 a	0.0030 ± 0.0015 a	3424.43 ± 28.95 a	3424.77 ± 5.86 a
	R2	6.43 ± 0.18 b	0.0055 ± 0.0012 a	3221.06 ± 192.29 a	3202.29 ± 207.37 a
Heading	BS	7.11 ± 0.13 a	0.0024 ± 0.0010 b	3531.04 ± 144.03 a	3537.76 ± 136.26 a
	R1	7.18 ± 0.02 a	0.0017 ± 0.0001 b	3708.30 ± 66.64 a	3681.90 ± 43.56 a
	R2	6.40 ± 0.09 b	0.0063 ± 0.0006 a	3165.68 ± 129.61b	3150.80 ± 137.51 b
Mature	BS	6.80 ± 0.37 a	0.0056 ± 0.0058 a	3244.54 ± 322.40 b	3246.05 ± 320.94 b
	R1	7.15 ± 0.04 a	0.0018 ± 0.0002 a	3729.68 ± 51.30 a	3755.11 ± 74.49 a
	R2	6.24 ± 0.15b	0.0074 ± 0.0016 a	3115.66 ± 98.94b	3122.62 ± 45.58b

Values with different letters indicate significant differences (analysis of variance; *p* < 0.05). BS, bulk soil; *R*1, rhizosphere soil; *R*2, rhizoplane soil.

### Soil bacterial community composition

**Figure 1:**
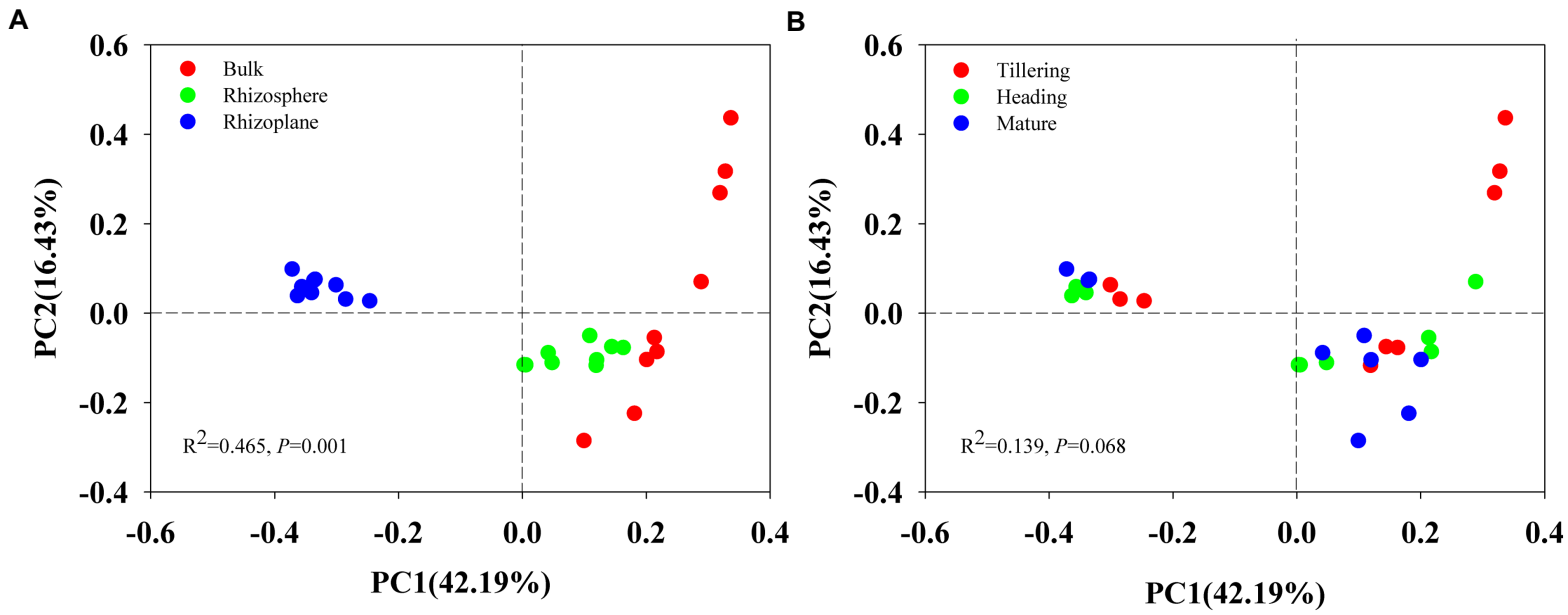
Principal Coordinates Analysis (PCoA) on Bray_Curtis dissimilarity demonstrated the differences of bacterial communities of 27 soil samples at two grouping patterns: **(A)** grouping pattern as bulk soil, rhizosphere and rhizoplane soil; **(B)** grouping pattern of tillering, heading, and mature stage.

Using permutational multivariate analysis of variance (PERMANOVA), root compartment niches had a substantial impact on the composition of the soil bacterial population (*r*^2^ = 0.465, *p* = 0.001) but had no effect on growth stage (*r*^2^ = 0.139, *p* = 0.068). Principal Coordinates Analysis (PCoA) also showed distinct bacterial community composition in different soils (bulk, rhizosphere and rhizoplane; Figure 1A), but bacterial communities of the tillering, heading and mature stage overlapped (Figure 1B), indicating that the largest source of variation in root-associated microbial communities is proximity to the root.

**Figure 2:**
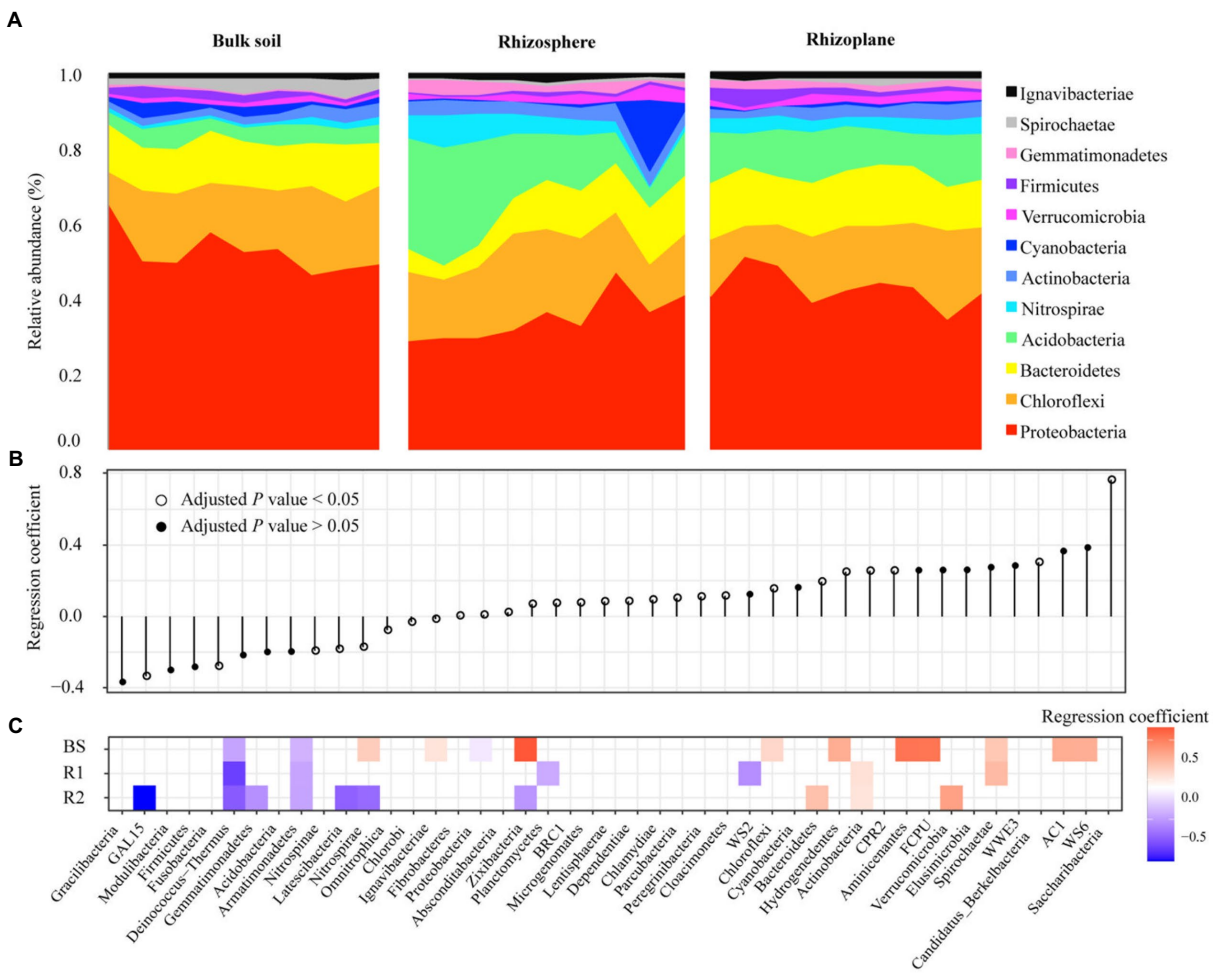
Shifts in the bacteria over time are associated with increasing and decreasing phyla. Bar plots of the top 12 phyla abundances over the course of the seasons in each compartment **(A)**. Beta regression coefficient estimates for bacterial phyla that are either increasing (above 0) or decreasing (below 0) in relative abundance in spatial distribution from the bulk to the rhizoplane soils **(B)**. Beta regression coefficient estimates for microbial phyla that are increasing (above 0) or decreasing (below 0) in relative abundance over the course of the growing stage in each compartment **(C)**.

Four bacterial phyla including Proteobacteria, Chloroflexi, Bacteroidetes, and Acidobacteria were predominant across all soil samples and 80.51% of the total bacterial sequences (Figure 2A). In addition, Actinobacteria, Nitrospirae, Verrucomicrobia, Cyanobacteria, Firmicutes, Gemmatimonadetes, Spirochaetae, and Ignavibacteriae were less abundant (relative abundance >0.1%) but were still identified in all soil samples. These bacterial phyla were significantly influenced by rhizocompartment throughout the growth stage of rice (Figure 2B). Of the 42 detectable phyla, we found 15 phyla with significantly different geographic distributions across the bulk and rhizoplane soils (Figure 2B). From the bulk to the rhizoplane soils, there were 9 phyla whose relative abundance considerably rose and 6 bacterial phyla whose relative abundance declined. Within each compartment, we conducted beta-regression to identify phyla whose relative abundance changed during the duration of the growth stage (Figure 2C). In rhizoplane soils, 3 bacterial phyla all increased, whereas 7 bacterial phyla consistently decreased during the growth stage. In rhizosphere soils, 2 bacterial phyla all increase, while 4 bacterial phyla consistently decreased during the growth stage. In bulk soils, 10 bacterial phyla all rose during the growth stage, while 3 bacterial phyla consistently decreased. In general, bulk soil had more phyla increased across the growing season than that of root related-compartments.

### Assembly processes of the bacterial communities

**Figure 3:**
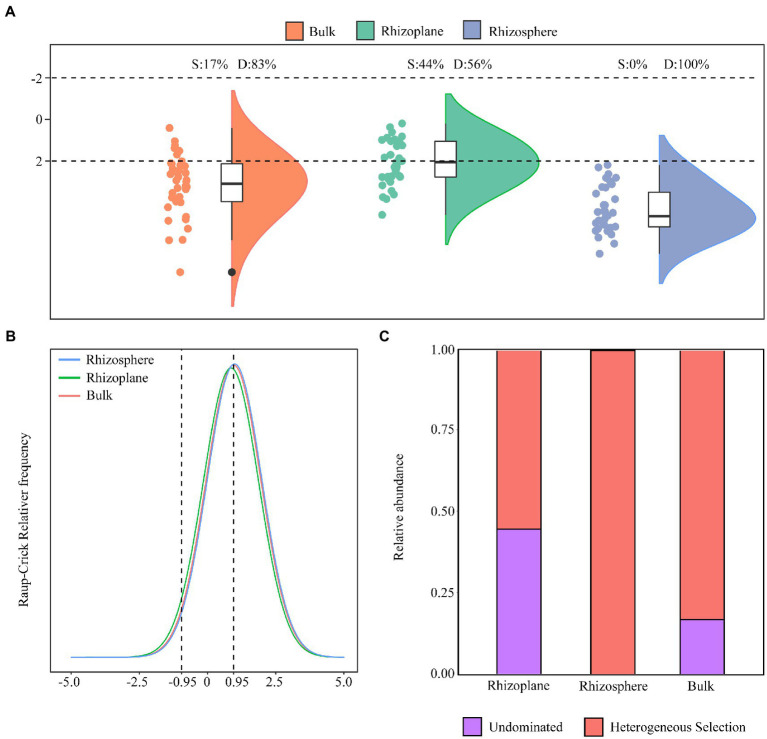
Deterministic and stochastic processes in bacterial assembly. Relative contribution of determinism and stochasticity on bacterial assembly based on the β-Nearest Taxon Index (βNTI) values **(A)**, Raup-Crick Relative frequency **(B)** and the relative importance of ecological processes **(C)**.

We calculated the beta nearest taxon index (NTI) of three compartments in order to distinguish between the deterministic and stochastic processes in community assembly along spatial distribution from the exterior to the surface of the root. βNTI values of the three groups were mainly above 2 (Figure 3A), always bulk soil (83%), rhizosphere (100%), and rhizoplane (56%). For the bulk and rhizosphere soils, higher relative deterministic process contribution primarily belongs to heterogeneous selection, while rhizoplane soil, the relative abundances of heterogeneous selection and undominated were similar (Figures 3B,C). Overall, deterministic mechanisms had a stronger impact on the bacterial community in the compartments connected to the roots.

### Co-occurrence networks of soil bacterial communities

**Figure 4:**
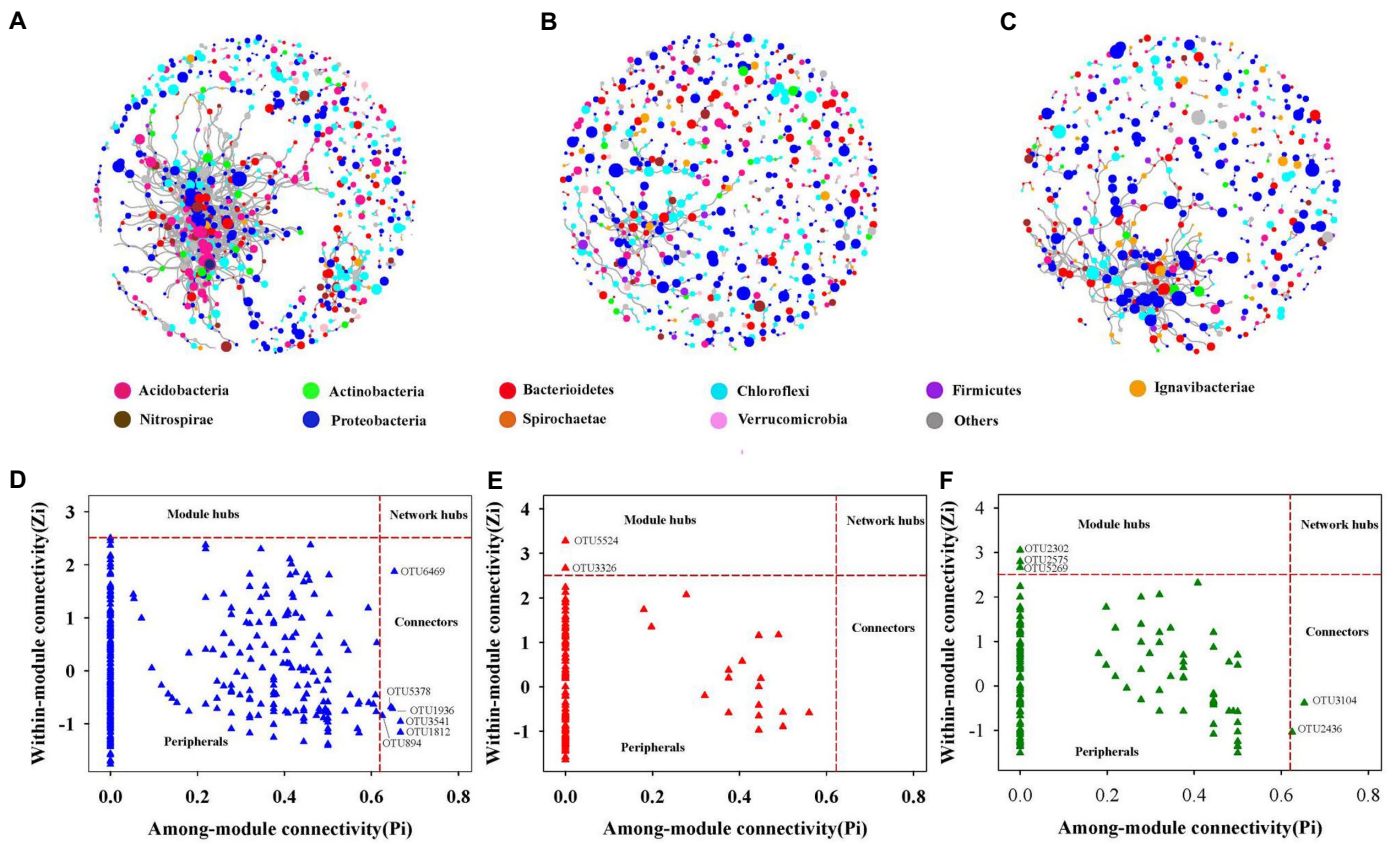
The co-occurrence network of soil bacterial community in bulk **(A)**, rhizosphere **(B)** and rhizoplane soils **(C)**, the size of each node is proportional to its relative abundance. Zi-Pi plots showing the distribution of soil bacterial OTUs based on their topological roles in bulk **(D)**, rhizosphere **(E)** and rhizoplane soil **(F)**.

Three sections of the soil bacterial community’s co-occurrence networks were examined Figures 4A–C and Table 2 lists the major topological characteristics. The network size was larger in bulk soils compared with rhizosphere and rhizoplane soils (Figures 4A–C), the number of nodes and links reflects this (Figures 4A–C; Table 2). The bulk soils networks were better connected and more complicated than rhizosphere and rhizoplane soils network (Figures 4A–C). The network topological qualities further supported this pattern, that is, the connectedness in bulk soils existed the highest, followed by rhizoplane and rhizosphere, while modularity exhibited opposite trend. The whole network is divided into many clusters according to phylum level features, rhizosphere soils was the one with the most clusters (186), considerably greater than bulk soils (135) and rhizoplane soils (130) (Table 2). Additionally, compared to rhizosphere and rhizoplane soils, bulk soil networks had a larger ratio of positive to negative links (Figures 4A–C; Table 2).

**Table 2:** Major topological properties of the co-occurrence networks of soil bacterial communities in BS, R1, and R2 treatments.

Topological properties	BS	*R*1	*R*2
No. of original OTUs	2,697	3,070	2,295
Total nodes	982	749	577
Total edges	3,633	725	729
Average degree	7.40	1.94	2.53
No. of clustersa	135	186	130
Average clustering coefficient	0.50	0.36	0.37
Average path distance	5.54	5.91	6.74
Modularity	0.61	0.93	0.80
Connectedness	0.28	0.03	0.17
Positive links/negative links	1.40	1.19	1.37

BS, bulk soil; *R*1, rhizosphere soil; *R*2, rhizoplane soil.

From the plot of Zi (a value measuring within-module connectivity) and Pi (a value measuring among-module connectivity), more module hub and connector were observed in bulk and rhizoplane networks than in rhizosphere (Figures 4D–F). Six keystone taxa were identified in bulk soils, belonged primarily to Proteobacteria, Verrucomicrobia, Chloroflexi, and Acidobacteria. Two keystone taxa were identified in rhizosphere soils, belonged primarily to Proteobacteria and Nitrospirae. Five keystone taxa were identified in rhizoplane soils, belonged primarily to Proteobacteria, Chloroflexi, Firmicutes, and Bacteroidetes (Figures 4D–F; Supplementary Table S4). Supplementary Table S4 shows the evolutionary classification of each module hub and connection.

**Figure 5:**
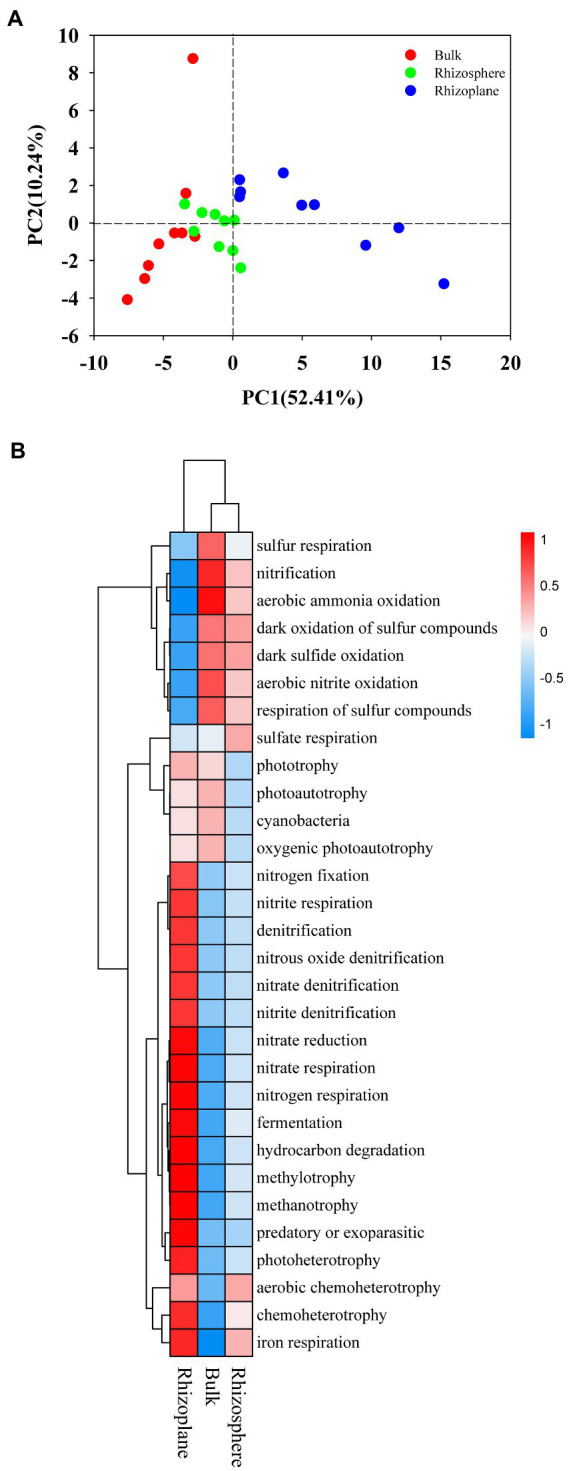
Principal Component Analysis (PCA) **(A)** and heatmap **(B)** of the soil bacterial community upon the functional groups predicted by FAPROTAX among all samples. BS, bulk soil; R1, rhizosphere soil; R2, rhizoplane soil.

### Soil bacterial community functional groups

The bacterial community in the soil was evaluated using FAPROTAX analysis, and 58 ecological types were predicted, with chemoheterotrophy, aerobic chemoheterotrophy, nitrification, and fermentation as the dominant functions. The PCA analysis was applied to visualize the differences among samples showed distinct bacterial functional profiles in different rhizocompartments (Figure 5A), demonstrating distinct communities with some overlap. Regarding the functional profile, the functional groups belonging to S and N cycle were more abundant in bulk and rhizosphere soils (*p* < 0.05), while functional groups belonging to C and Fe cycle were more abundant in rhizoplane soils (Figure 5B; Supplementary Table S5; *p* < 0.05).

## Discussion

Several studies indicate that microbial communities associated with plants have been discovered to be advantageous for plant growth and resistance against biotic and abiotic stresses (Van Syoc et al., 2022; Zhang et al., 2022). Therefore, understanding the spatial pattern and temporal evolution of the root-associated microbiota is essential for agroecosystem function (Edwards et al., 2015). Our findings in this study showed that root compartment niches, but not temporal variation, significantly impacted bacterial α-diversity indices. Although it has been repeatedly shown that the root microbiome changed as the plant shifted throughout its life cycle (Chaparro et al., 2014; Edwards et al., 2015; Dombrowski et al., 2016). Soil environments have been considered as a hot spot for studying the biodiversity of plant-associated microbiota (Compant et al., 2010). Sanaullah et al. (2016) showed that different enzyme systems exist at the plant–soil interface including the rhizosphere, rhizoplane soil, and bulk soil. Soil bacteria be more sensitive to changes in soil enzyme activity than changes in the rice life cycle (Gong et al., 2019). Root compartment niches appears to influence soil microbial diversity through its effect on soil enzymes. Mollisols is commonly fertile and productive with high content of organic materials (Liu et al., 2012), and the SOM content was 35.1 g·kg^−1^ in the present study. Moreover, nitrogen fertilizer applied in green and jointing stage, respectively. Consequently, the high SOM and N supplement could potentially lessen how plant growth stages affect bacterial diversity. Overall, we noticed similarities in patterns in alpha-diversity at tillering and heading stages (Table 1). The alpha-diversity was significantly lower in in rhizoplane soils than that of bulk and rhizosphere soils (Table 1). Cui et al. (2019) found that plant selects microorganisms and as a result, rhizosphere microbial community diversity is less than that of bulk soils.

Similar soil bacterial diversity, community structure responded to root compartment niches over the course of the growth stage. This finding verified the outcomes of Edwards et al. (2015) who demonstrated that the largest source of variance in the sampled microbial communities was described by the rhizocompartments. The root “filtration effect,” which allows plants to choose a microbiome in two steps, was assumed to be the cause of the variable alpha-diversity and community structure with root proximity (Dibbern et al., 2014). The transition from external to internal occupancy in the root comes first, followed by a general recruitment to the area around the root (Edwards et al., 2015). It is possible that the host plant will select against the declining variety of the bacteria associated with the roots or that they will finally be outcompeted by other taxa (Edwards et al., 2018). In our study, rhizoplane had a lower relative abundance of Acidobacteria (Figure 2B), which decreased monotonically by soil pH changes. Fan et al. (2017) demonstrated that Acidobacteria were depleted in rhizospheric soil. Conversely, advantageous microbes that enhance nutrient acquisition and combat pathogenic taxa (Bull et al., 2005; János, 2005), e.g., Bacteroidetes and Actinobacteria, had enriched in rhizosphere and rhizoplane soils.

Our findings demonstrating the same patterns of βNTI values of various root compartment niches suggest that deterministic processes tend to dominate community assembly (Figure 3). Regarding this project, Fan et al. (2017) reported that most bacterial community assemblages in bulk soil, loosely bound soil, and tightly bound soil in wheat crop fields tended to be dominated by deterministic mechanisms. Moreover, it is also interesting to note that when the bacterial Shannon diversity indices level is low, deterministic mechanisms frequently predominate during community assembly (Xun et al., 2019). Recent research proposed that with decreased biomass and a smaller population, the community is more susceptible to drift (a stochastic process) or founder effects (Evans et al., 2017). Nevertheless, dispersal, which is dependent on the local environment, can have a significant impact on the local dominance of stochastic or deterministic processes in an established community with saturated population or community size (Vellend et al., 2007). Hence, the decreased bacterial diversity from the exterior to the surface of the root may be attributed to environmental selection.

Biological communities’ ecological networks were lately widely used in plant and soil microbial ecology (Bastolla et al., 2009; Guan et al., 2020). Previous study has shown that microbial network complexity was impacted by farming systems, soil type, and ecological niches (Fan et al., 2017). Root compartment niches had an impact on the structure of the bacterial population in the soil as well as the pattern of bacterial co-occurrence. Our study showed that the network was more complexity in bulk soils compared with rhizosphere and rhizoplane soils (Figure 4), as evaluated by more nodes and stronger connections. This suggests that bacterial communities in bulk soils are more likely to be able to establish mutually beneficial communities and enhance resistance to external disturbances (Scheffer et al., 2012). In addition, more connected networks can use carbon more efficiently and improve nutrient exchange between various species (Morriën et al., 2017). This could mean that bacterial communities in bulk soil have more carbon-related energy sources, which is also reflected in bulk soils having more phyla increased. These findings are in line with those of Fan et al. (2017), who discovered that in wheat crop fields, bulk soil is more connected than tightly bonded and loosely bound soils. The distinction of topological among three compartments may be attributed the niche differentiation (Ma et al., 2015). In this sense, the interaction of soil physical–chemical properties and root-derived products, which shapes the niches and exerts niche pressures in the community assembly, has a significant impact on the observed niche-based selection of the rhizosphere microbiome (Mendes et al., 2014). Moreover, from the perspective of plants, community assembly in the rhizosphere is frequently seen as an increasingly plant-driven selection of microbial taxa from bulk soil to the rhizosphere and rhizoplane (Rüger et al., 2021). Therefore, a less complex network is anticipated in our study since the rhizosphere community is a subset of the bulk soil community (Mendes et al., 2014). Notably, rhizosphere soils had a considerably higher Shannon index than that of rhizoplane soils at all growth stages, and yet we observed a more complex network structure in rhizoplane soils. It has been demonstrated that the increase in the Shannon index of the bacterial community is accompanied by a more complex network structure (Pereira et al., 2021; Yang F. et al., 2022). Microbial taxa in the rhizoplane soil ecotone have strong interactions with each other, which may contribute to the complexity of the bacterial network in rhizoplane soils (Li F. Q. et al., 2022). In addition, rhizoplane soils are also capable of recruiting enriched stress-resistant bacteria to combat external environmental stresses (Huang et al., 2022). Indeed, the number of associations that the community’s taxa have with one another rather than the number of taxa in the community generally determines the complexity of the microbiome (Banerjee et al., 2019). Moreover, compared to bulk soils, the complexity of the co-occurrence network dropped considerably, while the number of negative co-occurrences in rhizosphere and rhizoplane soils increased, possibly indicating that these bacterial species are competing for resources (Fuhrman, 2009).

Keystone species are crucial to the microbiome and their absence would significantly change the composition of the microbiome (Berry and Widder, 2014; Banerjee et al., 2018). The Zi-Pi plot (Figures 4D–F) revealed that generalists inhabited a small percentage of modules, frequently less than 2% of all modules in soil bacterial networks (Barberan et al., 2012; Jiang et al., 2015). In the three networks, nodes’ roles also changed. For example, OTU 1936, OTU 894, OTU 5378, OTU 1812, and OTU 6469 were found to be specialists in rhizoplane and rhizosphere soils networks but generalists in bulk soils networks, indicating that networks were well organized and that root compartment niches could change the topological roles of specific OTUs and important microbial populations Moreover, of the taxa identified as keystone taxa in rhizosphere soils, *Nitrospira* (OTU3326) might be related in nitrifying, indicating to a possible increase in nitrification in the rhizosphere inches (Ehrich et al., 1995; Ahmed, 2011). These generalists bridged various nodes between various modules or inside their own modules (Ling et al., 2016), As a result, these generalists might be able to form relationships with other species and control the nitrogen cycle in Mollisols.

Like the community structure, distinct functional cluster were found among three compartments. Changes in bacterial community structure may be a reflection of modifications in how that structure functions (Qu et al., 2020). According to our findings, the group capable of chemoheterotrophy was the primary predictors of soil bacterial functional structure, then followed by the groups of aerobic chemoheterotrophy, nitrification, and fermentation. These functional groups were associated with the carbon and nitrogen cycle in the soil (Liang et al., 2019), suggesting that changes in the soil’s carbon and nitrogen pools may be the primary factor influencing the functional organization of soil bacteria (O’Donnell et al., 2001). Carbon and nitrogen cycles in the soil nutrient cycle are essential for sustainable agroecosystems (Enebe and Babalola, 2021). Our study found that the bacterial functional groups associated with the nitrogen cycle were enriched in bulk and rhizosphere soils, this may be due to variations in soil enzyme activity caused by changes in ecological niche. Yang et al. (2017) found that the activities of enzymes related to the nitrogen cycle (urease and protease) showed significant differences in bulk and rhizosphere soils by comparing the enzymatic activities of heavy metal contaminated bulk and rhizosphere soils in the Qinling region. Furthermore, the rhizoplane has been proved to be a specialized niche for some taxa (Edwards et al., 2015). The rhizoplane appears to play a key gating role in the process of microbiome acquisition in the root, as the initially recruited microorganisms are subjected to physically selective binding at the rhizoplane to the root interstitial (Edwards et al., 2015). Since rice cultivation is responsible for some degree of carbon loss (CH_4_ emission) from farmland, rhizoplane may recruit more functional groups related to the carbon cycle to maintain plant growth requirements. The functional groups sulfur and nitrogen cycling processes in the bulk soils had considerably higher abundances than was discovered in the rhizoplane soils. However, the functional groups involved in the cycling of carbon and iron were more abundant in rhizoplane soils, suggesting that niches shift structure of the bacterial community from sulfur and nitrogen utilizing bacteria to carbon and iron cycle involving bacteria of rice in Mollisols.

## Conclusion

Our work demonstrated that root compartment niches is the main factor affecting the structure of soil bacterial communities rather than rice life cycle, as evidenced by changes in bacterial diversity and composition. Proteobacteria, Chloroflexi, Bacteroidetes, and Acidobacteria may be the core taxa in bacterial communities and may be important in maintaining the networks of bacterial community interactions. In all three compartments, deterministic processes dominated the bacterial community assemblage, and all were dominated by heterogeneous selection. In comparison to rhizosphere and rhizoplane soils, bulk soils had a more sophisticated bacterial co-occurrence network, as evidenced by more nodes, more hubs, and stronger connections, which was mainly driven by compartment niches. In bulk soils, sulfur-and nitrogen-using bacteria predominated; in rhizoplane soils, however, there were greater abundances of bacteria involved in the cycling of carbon and iron. Overall, our data demonstrate the importance of the mechanism of ecological niche changes caused from the outside to the inside of roots in the structure of bacterial communities in Mollisols of Northeast China, rather than the rice life cycle. The rice life cycle may lead to changes in plant and soil metabolites, which requires the application of soil metabolomics to establish mechanistic links between soil metabolites and microbial communities, which still needs further exploration.

## Data availability statement

The datasets presented in this study can be found in online repositories. The names of the repository/repositories and accession number(s) can be found in the article/Supplementary material.

## Author contributions

KL, MS, and QL: methodology. QW and SG: data processing. KL, LS, and JG: literature review. KL and MS: writing–original draft preparation. JB and SG: writing–review and editing. JB: supervision. KL and JB: funding acquisition. All authors contributed to the article and approved the submitted version.

## Funding

The study was funded by the Scientific research business cost project of provincial scientific research institutes in Heilongjiang Province “Identification and database establishment of core germplasm resources of saline-alkali tolerant rice” (CZKYF2022-1-B011); Innovation project funded by Heilongjiang Academy of Agricultural Sciences(CX23ZD0); and National key R&D plan “Key technologies and demonstration of white soil barrier reduction and productivity improvement in Sanjiang Plain” (2022YFD1500800).

## Conflict of interest

The authors declare that the research was conducted in the absence of any commercial or financial relationships that could be construed as a potential conflict of interest.

## Publisher’s note

All claims expressed in this article are solely those of the authors and do not necessarily represent those of their affiliated organizations, or those of the publisher, the editors and the reviewers. Any product that may be evaluated in this article, or claim that may be made by its manufacturer, is not guaranteed or endorsed by the publisher.
