# Supplementary material for: Soil bacterial communities of paddy are dependent on root compartment niches but independent of growth stages from Mollisols of Northeast China
Source: Front Microbiol. 2023 Apr 14;14:1170611. doi: 10.3389/fmicb.2023.1170611 (PMC10140518; doi:10.3389/fmicb.2023.1170611)
Supplement: Supplementary file 1 [file Data_Sheet_1.docx]

**Supplementary Material**

**Supplementary Information S1.** Soil sampling procedure and compartment separation

**Supplementary Information S2.** PCR conditions

**Supplementary Table S1.** Two-way ANOVAs for the effects of root compartment niches (RC), temporal change (TC) and their interaction (RC×TC) on soil bacterial diversity and richness indices

**Supplementary Table S2.** The classification at each taxonomic level of the generalists.

**Supplementary Table S3.** One-way ANOVA examining the effects of root compartment niches on the abundance of functional groups.

**Supplementary Information S1.** Soil sampling procedure and compartment separation

Briefly, harvested roots were manually shaken to sampled bulk soil (~2 inches below the soil surface) and then placed into 50-ml Falcon tubes with 15 ml of sterile phosphate-buffered saline (PBS) solution. The rhizosphere soil was concentrated by centrifuging Falcon tubes for 30 seconds at 10,000 g. The supernatant was discarded leaving only the soil fraction behind. The roots designated for rhizoplane collection were placed in a Falcon tube with 15 ml PBS, and tightly adhering microbes at the root surface were removed using a sonication protocol. The roots in the Falcon tube were sonicated for 30 s at 50-60 Hz. The roots were then discarded, and the liquid PBS fraction was kept as the rhizoplane compartment. The rhizoplane compartment was concentrated in the same manner, except all 15 mL of the sample was concentrated in the same 2 mL tube using multiple centrifugations.

**Supplementary Information S2.** PCR conditions

For each sample, three independent amplification reactions were performed. The PCR mixture (final volume 20 μL) contained 2 μL of template DNA, 0.8 μL of each primer, 4 μL of 5 × FastPfu Buffer, 2 μL of 2.5 mM dNTPs, 4 μL of FastPfu Polymerase, and sterile double-distilled H2O. The amplification conditions consisted of an initial denaturation step at 95℃ for 2 min, followed by 25 cycles of denaturation at 95℃ for 30 s, annealing at 55℃ for 30 s, and elongation at 72℃ for 30 s. Cycling was completed by a final elongation at 72℃ for 5 min. During amplification, a negative control reaction lacking template DNA was included to check for experimental contamination. PCR products were detected by electrophoresis on a 2% agarose gel and purified using an AxyPrep DNA Gel Extraction Kit (Axygen Biosciences, Union City, CA, USA) and quantified using a QuantiFluor™-ST (Promega, USA), following the standard PCR procedure. The purified amplicons were pooled in equimolar amounts and paired-end sequenced (2 × 250) on an Illumina MiSeq platform at the Majorbio Corporation (Shanghai, China).

**Supplementary Table S1.** Two-way ANOVAs for the effects of root compartment niches (RC), temporal change (TC) and their interaction (RC×TC) on soil bacterial diversity and richness indices

| Variables | RC | |  | TC | |  | RC×TC | |
| --- | --- | --- | --- | --- | --- | --- | --- | --- |
|  | *F* | *P* |  | *F* | *P* |  | *F* | *P* |
| Shannon | 40.078 | **<0.001** |  | 3.417 | 0.055 |  | 1.799 | 0.173 |
| Simpson | 8.595 | **0.002** |  | 1.026 | 0.379 |  | .793 | 0.545 |
| Ace | 14.770 | **<0.001** |  | 3.221 | 0.064 |  | 1.949 | 0.146 |
| Chao 1 | 16.344 | **<0.001** |  | 3.220 | 0.064 |  | 2.186 | 0.112 |

**Supplementary Table S3.** One-way ANOVA examining the effects of root compartment niches on the abundance of functional groups.

| Functional groups | *F* | *P* |
| --- | --- | --- |
| Chemoheterotrophy | 14.19 | **0.00** |
| Aerobic chemoheterotrophy | 3.67 | **0.04** |
| Nitrification | 25.98 | **0.00** |
| Fermentation | 20.96 | **0.00** |
| Methylotrophy | 25.16 | **0.00** |
| Hydrocarbon degradation | 23.47 | **0.00** |
| Aerobic nitrite oxidation | 10.38 | **0.00** |
| Methanotrophy | 24.03 | **0.00** |
| Phototrophy | 0.93 | 0.41 |
| Aerobic ammonia oxidation | 52.11 | **0.00** |
| Iron respiration | 38.64 | **0.00** |
| Respiration of sulfur compounds | 7.72 | **0.00** |
| Photoautotrophy | 0.82 | 0.45 |
| Nitrate reduction | 17.12 | **0.00** |
| Cyanobacteria | 0.79 | 0.47 |
| Oxygenic photoautotrophy | 0.79 | 0.47 |
| Sulfate respiration | 0.62 | 0.55 |
| Nitrate respiration | 19.61 | **0.00** |
| Nitrogen respiration | 19.61 | **0.00** |
| Predatory or exoparasitic | 16.37 | **0.00** |
| Photoheterotrophy | 10.26 | **0.00** |
| Sulfur respiration | 3.70 | **0.04** |
| Nitrogen fixation | 5.04 | **0.02** |
| Dark oxidation of sulfur compounds | 8.68 | **0.00** |
| Dark sulfide oxidation | 9.15 | **0.00** |
| Nitrite respiration | 6.67 | **0.01** |
| Nitrate denitrification | 6.58 | **0.01** |
| Nitrite denitrification | 6.58 | **0.01** |
| Nitrous oxide denitrification | 6.58 | **0.01** |
| Denitrification | 6.58 | **0.01** |
